# Cardiac Fibrosis as a Dynamic Therapeutic Target: Overcoming Translational Barriers to Antifibrotic Therapy

**DOI:** 10.1016/j.jacbts.2026.101731

**Published:** 2026-09-22

**Authors:** Shuchishmita Maitra, Xander H.T. Wehrens, Svetlana Reilly

**Affiliations:** a Division of Cardiovascular Medicine, Radcliffe Department of Medicine, University of Oxford, Oxford, United Kingdom; b Department of Integrative Physiology, Cardiovascular Research Institute, Baylor College of Medicine, Houston, Texas, USA

**Keywords:** antifibrotic therapies, cardiovascular disease, fibrosis, translation

## Abstract

Cardiac fibrosis is a central feature of many cardiovascular diseases, contributing to myocardial stiffening, electrical dysfunction, and progression to heart failure. Despite its clinical importance, no therapy directly targeting myocardial fibrosis is currently established in practice. Advances in the understanding of cardiac fibroblast biology and profibrotic signaling pathways, including transforming growth factor-β and neurohormonal activation, have driven the development of multiple antifibrotic strategies in preclinical models, ranging from small molecules to gene- and cell-based approaches. However, translation into clinical benefit has remained limited, with existing evidence largely derived from early-phase studies that rely on indirect imaging or biomarker-based assessments. This review examines the mechanisms underpinning cardiac fibrosis and evaluates current antifibrotic strategies across key cardiovascular conditions from a translational perspective. We argue that translational failure arises not simply from ineffective therapies, but from a mismatch between the biological complexity of human fibrotic remodeling and the simplified paradigms underpinning current therapeutic development. We highlight key barriers to translation, including limitations of experimental models, fibroblast heterogeneity, temporal dependence of therapeutic efficacy, challenges in fibrosis assessment, and constraints in trial design. We further outline emerging strategies to bridge the translational gap.

## Highlights

• Cardiac fibrosis drives a range of cardiovascular diseases, although effective antifibrotic therapies remain limited.
• Fibrosis is heterogeneous and dynamically shaped by disease context, anatomical location, and fibroblast state.
• Translational failure reflects mismatches among human fibrotic remodeling, preclinical models, biomarkers, and trial design.
• Future success will require stage-specific, biomarker-guided strategies targeting active fibrosis alongside underlying disease drivers.

Cardiac fibrosis is a central feature of numerous cardiovascular diseases, including heart failure with preserved ejection fraction (HFpEF) and heart failure with reduced ejection fraction (HFrEF), postmyocardial infarction (MI) remodeling, and atrial fibrillation (AF).^1^, ^2^, ^3^, ^4^, ^5^ A 2025 global systematic review estimated that myocardial fibrosis is present in approximately one-third of individuals with cardiometabolic disease,^6^ whereas disease-specific studies suggest a substantially greater burden, with fibrosis identified in up to 93% of patients with HFpEF.^7^ Fibrosis disrupts myocardial structure, leading to increased ventricular stiffness and impaired electrical conduction, thereby exacerbating cardiac dysfunction.^8^ Across disease states, including AF, oxidative stress has been shown to contribute to structural remodeling processes that promote substrate formation.^9^ Despite its recognized pathologic importance, no therapy is currently approved specifically to reverse or prevent cardiac fibrosis.

The recent development of advanced experimental platforms, such as human induced pluripotent stem cell-derived models, engineered heart tissues, and in vivo systems, has enabled deeper insight into the cellular and molecular mechanisms driving cardiac fibrosis.^10^ Cardiac fibroblasts, once considered passive structural cells, are now recognized as dynamic regulators of myocardial homeostasis that respond to neurohormonal activation and inflammatory cues.^11^^,^^12^ Profibrotic pathways including transforming growth factor-β (TGF-β), renin-angiotensin-aldosterone system (RAAS) activation, immune-mediated signaling, and changes in extracellular matrix (ECM) stiffness and composition have emerged as central drivers of fibroblast activation and myofibroblast differentiation.^13^, ^14^, ^15^, ^16^, ^17^ Additional endocrine and paracrine signaling pathways have also been shown to directly regulate fibroblast activation and fibrotic remodeling.^18^^,^^19^

These advances have stimulated the development of numerous antifibrotic strategies ranging from small molecules and biologics to gene-, cell-, and RNA-based approaches.^20^ Although many interventions demonstrate robust antifibrotic effects in experimental systems, few have convincingly reduced fibrosis or improved meaningful cardiovascular outcomes in patients.^20^ This translational gap reflects the heterogeneity and context-dependent nature of human fibrotic remodeling, limitations in imaging and biomarker-based patient stratification, and the inability of many experimental models and therapeutic frameworks to capture disease-, stage-, and cell-state-specific biology.^12^^,^^20^^,^^21^ Fibrosis should, therefore, be viewed not as a static scar burden, but as a dynamic and spatially heterogeneous process involving evolving fibroblast states, immune interactions, and ECM remodeling.

Against this background, this narrative review provides a conceptually structured synthesis of the current understanding of cardiac fibrosis, with particular emphasis on the biological and clinical factors that may explain the persistent failure of antifibrotic therapies to translate from experimental models into clinical practice. We integrate mechanistic, preclinical, and clinical evidence to contextualize antifibrotic development within the heterogeneity of fibrotic phenotype, underlying cardiovascular disease, anatomical location, fibroblast state, and stage of remodeling. We first consider the heterogeneous forms and biological mechanisms of cardiac fibrosis before discussing representative antifibrotic strategies and the major translational barriers that have emerged across cardiovascular disease settings. By bringing together these complementary lines of evidence, we highlight recurring biological and clinical themes that may account for inconsistent therapeutic translation and identify emerging concepts that could guide more precise antifibrotic strategies. A central premise of this review is that translational failure reflects not simply the absence of effective therapies, but a mismatch between the biological complexity of human fibrotic remodeling and the simplified therapeutic frameworks that have guided antifibrotic development.

## Biology of Cardiac Fibrosis: Therapeutic Rationale

Cardiac fibrosis is characterized by the excessive accumulation of fibrous proteins in the ECM, predominantly type I and type III collagens.^22^, ^23^, ^24^ Although physiologic ECM turnover is essential for structural integrity, persistent or dysregulated ECM deposition leads to myocardial stiffening, impaired ventricular relaxation, and disrupted electrical coupling between cardiomyocytes (Figure 1), thereby contributing to both mechanical dysfunction and arrhythmogenesis,^8^^,^^25^, ^26^, ^27^, ^28^ making fibrosis both a central driver of cardiovascular disease and a therapeutic target. The following sections examine cardiac fibroblasts as the principal effector cells of fibrosis, the major pathways regulating their activation, and the importance of fibroblast heterogeneity in determining fibrotic responses.

**Figure 1:**
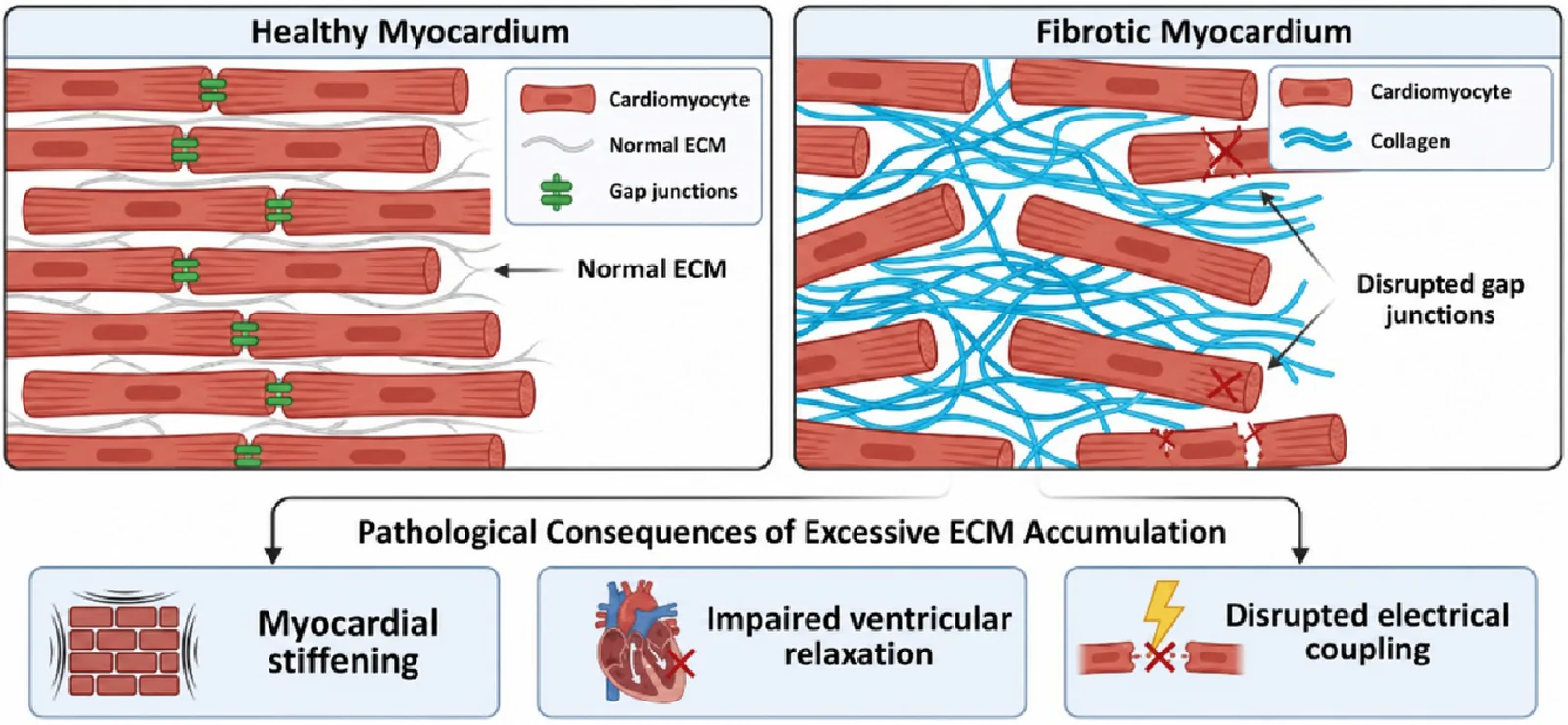
Cardiac Fibrosis Disrupts Myocardial Structure and Function Healthy myocardium exhibits organized cardiomyocytes with intact cell-cell connections (including gap junctions) embedded within a balanced extracellular matrix (ECM) that preserves normal tissue architecture and electrical conduction. In fibrosis, excessive collagen deposition and expansion of the ECM disrupt myocardial structure and gap junction organization, leading to impaired electrical coupling, altered mechanical function, and pathologic cardiac remodeling.

### Heterogeneous forms and temporal evolution of cardiac fibrosis

Cardiac fibrosis is not a single pathologic entity but comprises distinct forms, including interstitial/perivascular and replacement fibrosis, which arise through different pathophysiological processes.^1^ Replacement fibrosis develops in response to cardiomyocyte loss, whereas reactive fibrosis occurs with limited myocyte death and may involve the interstitial or perivascular compartments.^1^ Microscarring describes small focal areas of replacement fibrosis that can coexist with diffuse interstitial or perivascular fibrosis, as demonstrated histologically in pressure-overload states and severe aortic stenosis.^29^^,^^30^ Accordingly, a distinction can be made between adaptive fibrosis, which supports tissue repair and preservation of structural integrity after injury, and pathologic fibrosis, which becomes maladaptive and contributes to progressive cardiac dysfunction. Following acute injury, reparative or replacement fibrosis can be an adaptive response, as exemplified by scar formation after MI, where ECM deposition is required to mechanically stabilize necrotic tissue.^31^ In contrast, reactive fibrosis arises through different pathologic processes and may progressively extend from perivascular to interstitial compartments, as occurs during pressure overload.^31^ Such pathologic progression may be reinforced by increasing ECM stiffness, which enhances fibroblast activation and proliferation and, through mechanotransduction pathways, establishes a positive-feedback loop that perpetuates inflammation and fibrosis.^32^ In addition, the ECM is not simply a structural consequence of fibrosis, but an active regulator of cellular behavior that stores and releases growth factors, cytokines, and bioactive peptides.^33^ During remodeling, proteolytic processing of the ECM generates and releases bioactive matrix-derived fragments.^34^ Recently, the collagen VI-derived matrikine endotrophin has been shown experimentally to stimulate type I collagen synthesis in human cardiac fibroblasts,^35^ demonstrating that ECM-derived fragments can directly influence profibrotic cellular responses. Such fragments may, therefore, have potential value as mechanistic mediators, biomarkers, or therapeutic targets.

The mechanisms and consequences of fibrosis therefore vary with the nature of the underlying injury, the tissue or anatomical compartment involved, and the pathologic context.^36^ Accordingly, fibrosis arising in different cardiovascular disease settings should not be assumed to be biologically equivalent. This heterogeneity has important therapeutic implications, as the efficacy and safety of antifibrotic strategies are likely to depend on the fibrotic phenotype, disease context, and timing of intervention.

### Cardiac fibroblasts and myofibroblast activation

Cardiac fibroblasts represent the principal cellular mediators of myocardial fibrosis.^11^ In fibrotic diseases, resident cardiac fibroblasts differentiate into myofibroblasts, a phenotypically distinct state characterized by increased proliferative capacity and enhanced ECM synthesis, including increased expression of structural collagens and matricellular proteins.^37^^,^^38^ Importantly, myofibroblasts are largely absent in the healthy adult heart, as fibroblasts remain in a quiescent state.^39^ These observations highlight a central translational challenge: fibroblast activation is not a binary pathologic process, but a spectrum of multiple context-dependent states, raising the risk that nonselective antifibrotic strategies may disrupt adaptive repair while incompletely suppressing pathologic fibrosis. This concept has driven significant interest in fibroblast- and myofibroblast-specific antifibrotic strategies.^24^^,^^40^ The dynamic and context-dependent nature of fibroblast activation is illustrated in Figure 2.

**Figure 2:**
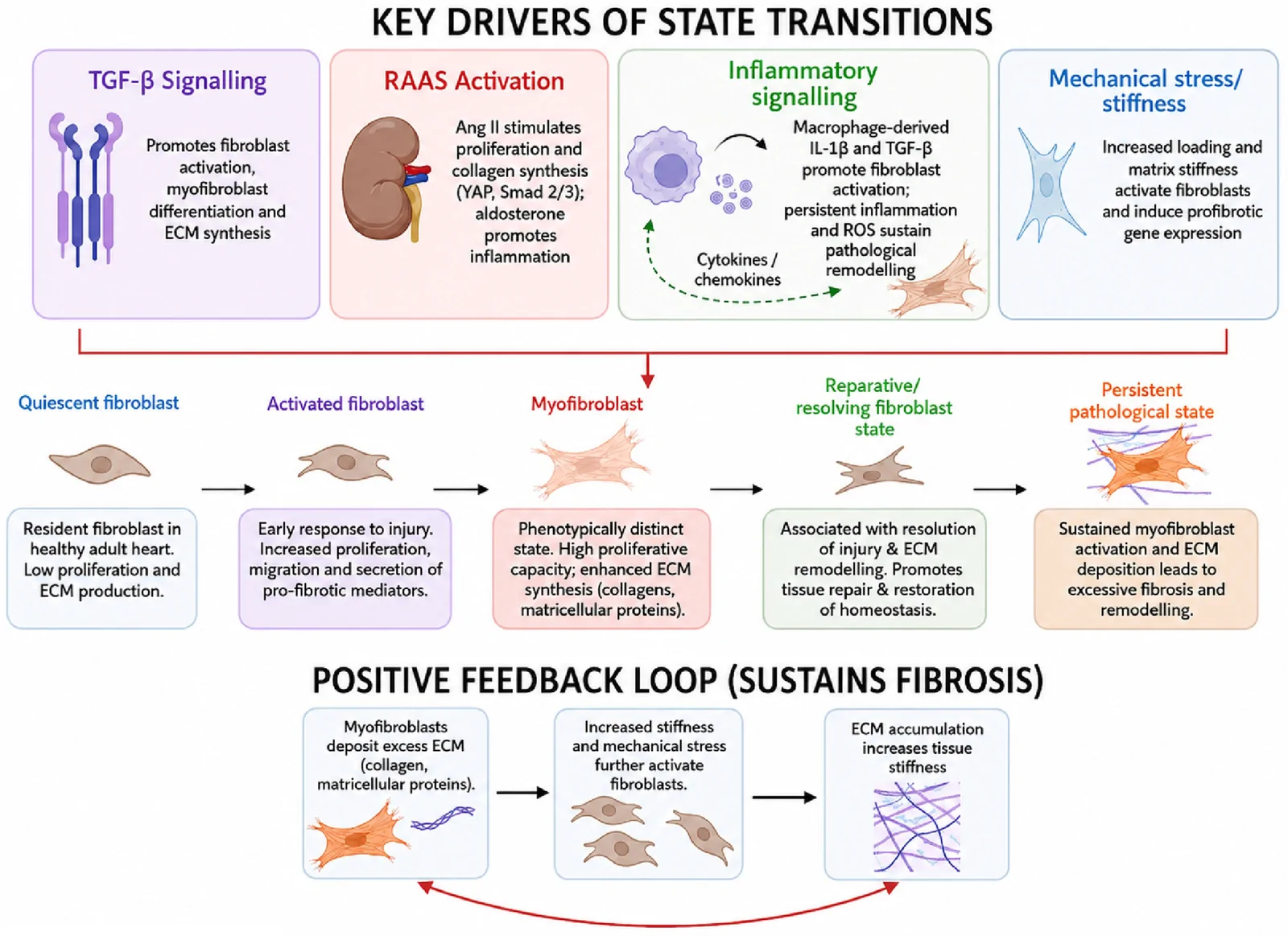
Fibroblast State Transitions in Cardiac Fibrosis Quiescent fibroblasts undergo activation in response to TGF-β, RAAS, inflammatory signaling, and mechanical stress, giving rise to myofibroblasts that promote extracellular matrix deposition. Activated fibroblasts may subsequently adopt reparative/resolving, persistent profibrotic, or senescent states depending on disease context. Extracellular matrix accumulation increases tissue stiffness, creating a positive feedback loop that further drives fibroblast activation and fibrosis. Ang II = angiotensin II; ECM = extracellular matrix; IL-1β = interleukin-1 beta; RAAS = renin-angiotensin-aldosterone system; ROS = reactive oxygen species; Smad2/3 = mothers against decapentaplegic homolog 2/3; TGF-β = transforming growth factor-beta; YAP = yes-associated protein.

### Fibroblast heterogeneity as a mechanistic determinant

Cardiac fibroblasts exhibit spatial heterogeneity, with distinct fibroblast states occupying different anatomical niches within the human heart.^41^ Recent single-cell, spatial transcriptomic, and multi-omic studies have revealed marked heterogeneity of cardiac fibroblast populations across anatomical regions and disease states. Single-cell and single-nucleus profiling of the human heart has revealed region-specific fibroblast populations and distinct atrial and ventricular cell-cell interaction networks,^42^ whereas heart failure is accompanied by marked diversification into disease-associated fibroblast states.^43^ Following myocardial injury, this heterogeneity becomes spatially organized, with profibrotic collagen triple helix repeat-containing 1^+^ fibroblasts emerging within the infarct scar^44^ and broader cell- and histology-specific transcriptional programs associated with fibrosis and advanced heart failure.^45^ Spatial multi-omic profiling of human MI has further identified disease-specific fibroblast states within distinct anatomical zones of injury, repair, and remodeling, highlighting the spatial and temporal heterogeneity of fibroblast responses following ischemic injury.^46^ Integration of single-cell transcriptomic data sets across diverse cardiac pathologies has further revealed both conserved and context-dependent fibroblast states, including similarities in fibroblast identities and activation dynamics between ischemic and pressure-overload cardiomyopathy.^47^ This heterogeneity complicates therapeutic targeting, as selective inhibition of pathogenic fibroblast populations is limited by overlapping markers, pathway pleiotropy, and challenges in cell-specific delivery.^11^ Single-cell studies indicate that cardiac fibroblasts include subsets with potentially antifibrotic properties; for example, a murine post-MI myofibroblast subcluster was enriched for CCN5/Wisp2, Sfrp1/2, and Htra1/3, consistent with attenuation of profibrotic growth factor signaling.^48^ These findings suggest functional heterogeneity among cardiac fibroblasts, with distinct populations contributing to either adaptive repair or pathologic remodeling. The principal signaling pathways and mechanical cues that regulate fibroblast activation and ECM deposition are summarized in Figure 3.

**Figure 3:**
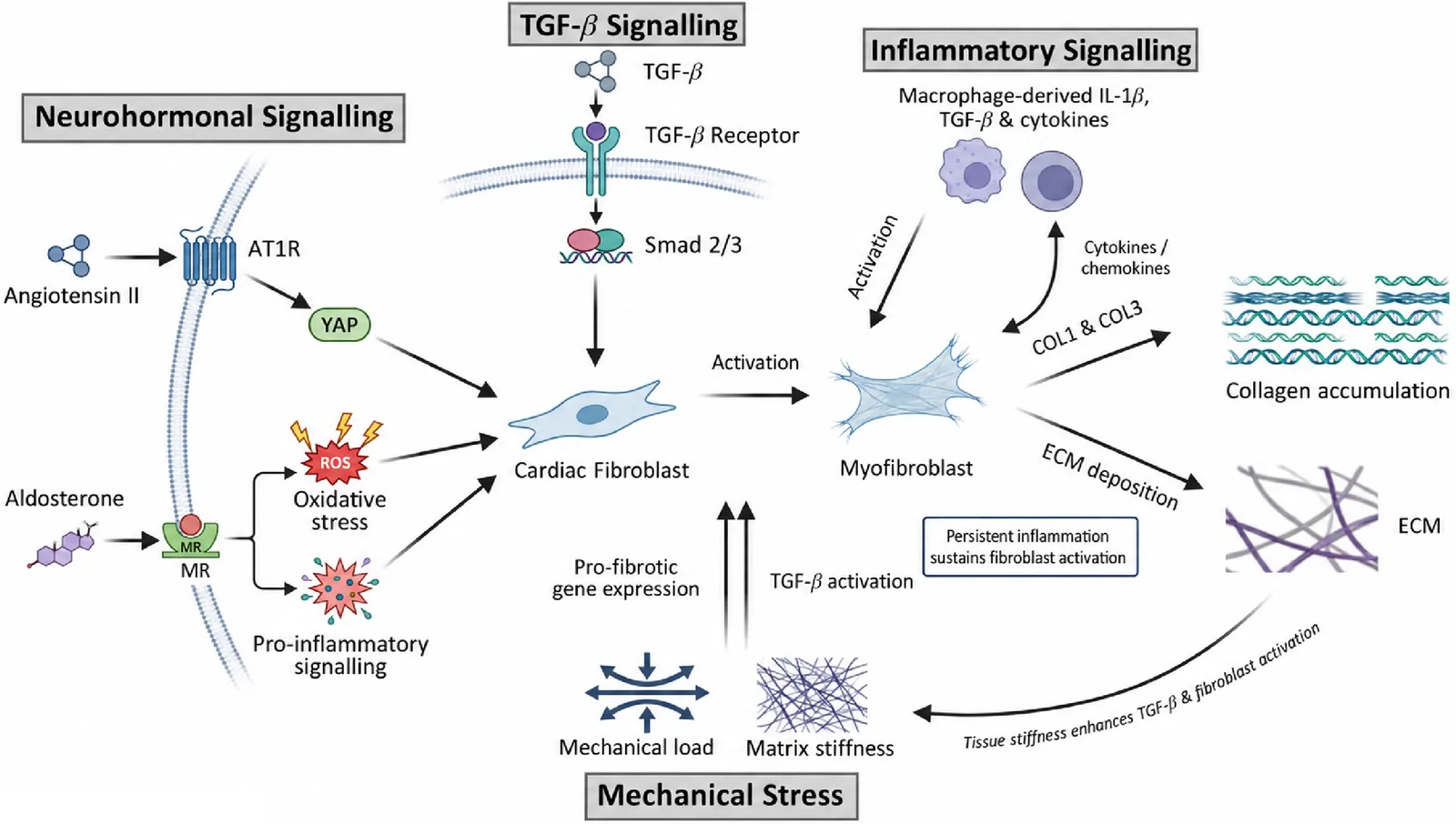
Key Signaling Pathways in Cardiac Fibrotic Remodeling Transforming growth factor-beta (TGF-β), neurohormonal, inflammatory, and mechanical signals promote fibroblast activation, increasing collagen and extracellular matrix (ECM) deposition. ECM accumulation enhances matrix stiffness and TGF-β activation, forming a positive-feedback loop that sustains fibrosis. AT1R = angiotensin II type 1 receptor; COL1 = collagen type I; COL3 = collagen type III; MR = mineralocorticoid receptor; SMAD2/3 = SMAD family member 2/3; other abbreviations as in Figure 2.

### TGF-β signaling

Among profibrotic pathways, TGF-β signaling is widely regarded as a central regulator of cardiac fibrosis.^13^ Through canonical Smad-dependent and noncanonical pathways, TGF-β promotes fibroblast activation, myofibroblast differentiation, and ECM synthesis.^49^, ^50^, ^51^ In the atria, TGF-β signaling promotes fibroblast activation, myofibroblast differentiation, and collagen-rich ECM deposition, contributing to structural remodeling and conduction heterogeneity that facilitate the development and persistence of AF.^52^

Consequently, experimental evidence reveals that TGF-β receptor I inhibition markedly attenuates fibroblast activation and collagen synthesis.^53^ Although TGF-β is a central profibrotic regulator, its broader roles in tissue homeostasis complicate therapeutic targeting and contribute to a narrow therapeutic window.^54^ These observations illustrate a broader challenge in antifibrotic drug development: the pathways most strongly implicated in fibrosis are often also fundamental to physiologic repair processes.

### Neurohormonal signaling

Neurohormonal activation, particularly through the RAAS, promotes fibroblast activation, collagen synthesis, and ECM remodeling.^55^, ^56^, ^57^, ^58^, ^59^ Angiotensin II promotes cardiac fibrosis by directly stimulating collagen synthesis in cardiac fibroblasts and activating profibrotic Smad signaling,^60^^,^^61^ whereas aldosterone further amplifies fibrotic remodeling through proinflammatory signaling, including activation of the interleukin 6 (IL-6) trans-signaling pathway^62^ and activation of nuclear factor κB–dependent profibrotic signaling in cardiac fibroblasts.^63^

### Inflammatory signaling and immune–fibroblast crosstalk

Inflammatory signaling regulates fibroblast behavior and ECM turnover following cardiac injury.^64^ Activated immune cells, particularly C-C motif chemokine receptor 2^+^ monocytes and macrophages, release inflammatory mediators such as IL-1β that promote cardiac fibroblast activation and subsequent fibrotic remodeling.^65^ Conversely, activated cardiac fibroblasts secrete cytokines and chemokines that promote immune-cell recruitment and modulate macrophage phenotype during cardiac remodeling.^66^^,^^67^ Immune–fibroblast interactions are stage dependent, with early proinflammatory signaling favoring M1 macrophage responses, followed by a TGF-β1-dominated reparative phase that promotes M2 polarization and tissue repair,^66^ whereas persistent inflammatory signaling can sustain fibroblast activation and pathologic fibrosis.^68^ Importantly, redox-dependent inflammatory pathways contribute to cardiac remodeling, with studies in AF demonstrating stage-specific changes in myocardial nicotinamide adenine dinucleotide phosphate oxidase–derived reactive oxygen species, suggesting that oxidative stress may act upstream of fibrotic remodeling processes.^9^ In addition, cardiomyocyte-derived paracrine signaling has emerged as an important regulator of fibroblast activation and fibrotic remodeling, further emphasizing the influence of cardiomyocytes on fibroblast behavior during cardiac remodeling.^18^ This suggests that profibrotic pathways do not operate in isolation but rather form an interconnected network and converge on fibroblast activation. This biological redundancy may partly explain why targeting individual signaling pathways has yielded limited translational success.

### Mechanical stress

Mechanical cues within the myocardial microenvironment are increasingly recognized as important regulators of fibroblast activation.^69^ Increased myocardial loading and matrix stiffening promote profibrotic gene expression and ECM accumulation, generating positive feedback loops that sustain fibrosis even after the initiating stimulus has resolved.^70^, ^71^, ^72^ For example, matrix stiffness and mechanotransduction contribute to cardiac fibroblast activation, with mechanically activated yes-associated protein signaling co-operating with TGF-β1 to promote profibrotic matrix remodeling.^73^ The integration of mechanical, inflammatory, and neurohormonal signals could complicate therapeutic targeting.

## Preclinical Antifibrotic Strategies

Although mechanistically diverse, preclinical antifibrotic strategies can be broadly understood within 3 conceptual strategies: inhibition of central profibrotic signaling pathways, modulation of fibroblast phenotype and activity, and targeting of ECM stabilization. Despite differences in approach, a common limitation is that these strategies largely focus on single nodes within a highly redundant fibrotic network. Multiple antifibrotic strategies have been investigated in experimental studies, including targeting profibrotic signaling pathways such as TGF-β and RAAS, regulating fibroblast activity, and investigating US Food and Drug Administration–approved drugs for antifibrotic effects.^10^^,^^54^^,^^74^ Despite encouraging antifibrotic effects reported in experimental models, clinical translation remains limited.^10^^,^^75^ Taken together, these approaches demonstrate that robust antifibrotic effects can be achieved in controlled experimental systems; however, their limited convergence on shared, translatable mechanisms underscores an important current disconnect between experimental efficacy and clinical applicability.

### Targeting TGF-β signaling

Targeting TGF-β signaling has been widely explored in preclinical studies of cardiac fibrosis. Preclinical studies demonstrate that modulation of TGF-β signaling can attenuate fibrotic remodeling;^76^ however, the requirement for such targeted approaches underscores the limitations of nonselective TGF-β blockade. Although some findings derive from noncardiac fibrosis models, they support a conserved role for TGF-β1 in fibrogenesis. Importantly, emerging evidence highlights marked context- and isoform-specific effects, with cardiomyocyte-derived TGFB3 attenuating fibrosis by restraining TGFB1-SMAD3 signaling.^77^

TGF-β signaling is required for tissue repair and scar integrity following MI, with disruption increasing the risk of adverse remodeling and cardiac rupture.^78^ This narrow therapeutic window requires careful balancing of antifibrotic efficacy against preservation of essential repair mechanisms. Similarly, disruption of individual components within the TGF-β axis can yield paradoxical effects; for example, knockdown of TIMP1 enhances fibroblast proliferation and augments TGF-β/Smad signaling in vitro.^79^ Moreover, the importance of TGF-β1 in immune homeostasis is illustrated by genetic loss-of-function studies in which TGF-β1 deficiency produces severe multifocal inflammatory disease,^80^ highlighting a potential consequence of indiscriminate pathway suppression. Global inhibition of TGF-β signaling could impair wound healing and provoke adverse effects,^81^ highlighting the need for more selective or context-dependent modulation.

### Neurohormonal modulation approach

Experimental studies have demonstrated that RAAS activation, particularly Ang II signaling, directly promotes cardiac fibroblast collagen synthesis and engages profibrotic Smad signaling,^60^^,^^61^ providing a mechanistic basis for RAAS-directed antifibrotic strategies. Mineralocorticoid receptor (MR) antagonists represent one of the most successfully translated neurohormonal therapies, with the landmark RALES trial establishing the clinical benefit of spironolactone in severe heart failure.^82^ More recently, nonsteroidal MR antagonists such as finerenone have demonstrated enhanced antifibrotic potential in preclinical models. In cardiac fibroblasts and Rac1-driven murine models, finerenone suppresses profibrotic signaling pathways, including TGF-β, resulting in reduced collagen deposition and attenuation of structural remodeling.^83^ Consistent with these findings, finerenone also reduces fibrosis and improves cardiac function in metabolic disease models, including type 2 diabetic rats.^84^ Notably, emerging evidence indicates that finerenone may exert antifibrotic effects through additional mechanisms beyond direct fibroblast modulation, including the attenuation of sodium accumulation and suppression of macrophage-mediated inflammation.^85^ This contrasts with earlier models of MR signaling that primarily emphasized direct MR-mediated fibroblast activation,^86^ highlighting a broader immunometabolic and microenvironmental role for MR antagonism in fibrotic remodeling. However, the evidence remains largely derived from preclinical systems, and differences in disease context, model design, and mechanistic endpoints complicate direct comparison and limit conclusions regarding clinical translatability.

### Small molecule antifibrotic agents

Small molecule antifibrotic agents originally developed for noncardiac fibrotic diseases have been explored in cardiac fibrosis, with compounds such as pirfenidone and nintedanib demonstrating antifibrotic effects in experimental models.^87^^,^^88^ Pirfenidone demonstrated selective antifibrotic effects in a 3-dimensional tissue-engineered human cardiac fibrosis model, reducing periostin and collagen-associated markers, although it did not reverse all TGF-β1-induced profibrotic changes.^88^ Nintedanib significantly attenuated myocardial fibrosis, reduced collagen gene expression, and preserved cardiac function in a pressure overload murine model.^87^ However, evidence remains largely preclinical, and the extent of fibrosis reversal is often incomplete.

More recently, attention has shifted toward targeting specific molecular drivers of fibrotic remodeling. LOXL2 is upregulated in diseased human myocardium and contributes to collagen cross-linking and fibroblast activation, whereas genetic or pharmacologic inhibition attenuates cardiac fibrosis in experimental models.^89^ More recently, the small-molecule LOXL2 inhibitor SNT-5382 reduced fibrosis in an MI model while demonstrating sustained clinical target engagement.^90^ In parallel, advances in computational drug discovery have enabled the identification of novel antifibrotic targets such as TRAF2- and NCK-interacting kinase.^91^ The small-molecule inhibitor INS018_055, developed using an artificial intelligence-driven pipeline, demonstrated antifibrotic and anti-inflammatory effects across multiple in vivo models.^91^ However, most evidence remains derived from experimental or noncardiac fibrosis models, and uncertainties regarding cardiac specificity, long-term efficacy, and safety continue to limit clinical translation. Collectively, these findings highlight the promise of small-molecule antifibrotic therapy, although clinical validation remains limited.

### Targeting matricellular proteins and extracellular mediators

Matricellular proteins such as galectin-3 have been investigated as targets in preclinical cardiac disease models. Preclinical studies using a direct protein inhibitor of galectin-3 demonstrated attenuation of post-MI fibrosis, accompanied by improved ventricular function and reduced adverse remodeling.^92^ Mechanistically, these effects were driven by suppression of profibrotic signaling pathways and inhibition of M2 macrophage polarization,^92^ highlighting the role of galectin-3 in coordinating immune-fibrotic crosstalk. Complementing this, indirect modulation of galectin-3 has shown antifibrotic potential. Chlorogenic acid reduced cardiac hypertrophy and fibrosis in an isoproterenol-induced model, with effects linked to down-regulation of galectin-3 expression and subsequent suppression of collagen-associated genes such as COL1A1.^93^ Notably, genetic manipulation of galectin-3 confirmed its central role in mediating these effects, as knockdown attenuated, whereas overexpression exacerbated, fibrotic and hypertrophic responses.^93^ Moreover, preclinical studies report that another matricellular protein, periostin, is upregulated in patients with heart failure and in post-MI remodeling, with ablation of periostin resulting in reduced cardiac dysfunction, injury, and fibrosis.^94^

Recent work further strengthens periostin as a fibrosis-relevant extracellular mediator but also adds an important complication: its role may be context- and sex-dependent. Preclinical models indicate that periostin-mediated remodeling exhibits sex-specific differences, with periostin-dependent fibrotic responses observed in male but not female mice.^95^ This highlights a potential limitation of broadly targeting periostin, as its role may vary depending on biological context and may also be linked to adaptive repair processes following injury.

### Epigenetic and microRNA-based approaches

Recent preclinical studies demonstrate that fibroblast activation and profibrotic gene expression are tightly regulated by epigenetic mechanisms, with chromatin remodeling factors such as SRCAP and KAT5 identified as important regulators of myofibroblast differentiation.^96^ Functional studies show that these chromatin complexes facilitate the activity of profibrotic transcription factors, whereas pharmacologic inhibition of KAT5 attenuates fibrotic responses in human cardiac fibroblasts,^96^ highlighting epigenetic control as a critical layer governing fibrotic remodeling. More recently, the histone acetyltransferase inhibitor anacardic acid attenuated postinfarction fibrosis in a murine model of permanent left anterior descending coronary artery ligation.^97^ Mechanistically, anacardic acid suppressed H3K9ac-dependent transcriptional activation of profibrotic genes and inhibited p38/JNK–GATA3 signaling, resulting in reduced fibroblast proliferation, migration, and myofibroblast differentiation.^97^ These findings support epigenetic modulation as a promising antifibrotic strategy; however, the pleiotropic nature of these pathways and uncertainties regarding target specificity continue to present translational challenges.

In parallel, microRNA-based approaches represent an additional regulatory layer. MicroRNA-based approaches exemplify the complexity of post-transcriptional regulation in cardiac fibrosis, with individual microRNAs exerting context-dependent pro- or antifibrotic effects across different cellular and disease settings.^98^ In AF, alterations in both circulating and tissue-derived microRNAs have been demonstrated in primary human studies, with circulating miR-21 associated with the extent of left atrial low-voltage substrate and tissue miR-31 implicated mechanistically in atrial remodeling.^99^^,^^100^ These observations highlight the potential of microRNAs as both mechanistic regulators and biomarkers of fibrotic remodeling, although their context-dependent effects and challenges in targeted delivery remain major barriers to clinical translation. A summary of these findings is presented in Table 1.

**Table 1:** Summary of Recent Preclinical Studies Investigating MicroRNA-Based Approaches for Targeting Cardiac Fibrosis

MicroRNA Candidate	Proposed Strategy	Study Design	Main Fibrosis-Related Finding	Mechanistic Target/Axis	Key Limitation
miR-129-5p	Upregulation (miRNA mimic)^101^	Human cardiac tissue + Ang II mouse model + primary cardiac fibroblasts^101^	Attenuates cardiac fibrosis and calcification; improves systolic and diastolic function; suppresses fibroblast activation^101^	miR-129-5p → SOX9/ASPN → β-catenin signaling regulating fibroblast plasticity^101^	Ang II model-specific; delivery and long-term translation not established
miR-424/322	Upregulation (miRNA mimic)^102^	Ang II–induced mouse model + human cardiac fibroblasts + knockout model^102^	Attenuates cardiac fibrosis and hypertrophy; knockout exacerbates remodeling^102^	NFATc3 → miR-424/322 → furin (negative feedback regulation)^102^	Ang II–specific model; limited pathway breadth; translational delivery not addressed
miR-512-3p	Upregulation (miRNA mimic)^103^	ISO-induced mouse model + Ang II–stimulated fibroblasts + target validation^103^	Attenuates myocardial fibrosis, fibroblast activation, and improves cardiac function^103^	miR-512-3p → SYK inhibition → reduced fibroblast activation and ECM production^103^	Limited model diversity; no translational delivery validation
miR-15a-5p	Inhibition (proposed; antagomir)^104^	Ang II mouse model + cardiomyocyte-fibroblast coculture + exosome analysis^104^	Promotes fibroblast activation and collagen production via intercellular signaling^104^	Exosomal miR-15a-5p → reduced DYRK2 → NFAT activation → fibrosis^104^	Primarily mechanistic; limited therapeutic validation; Ang II–specific model
miR-210	Inhibition (proposed)^105^	TAC-induced pressure overload mouse model + fibroblast assays^105^	Promotes fibrosis, inflammation, and adverse remodeling; overexpression worsens cardiac dysfunction^105^	Hypoxia-responsive miRNA-210 integrating inflammatory signaling and fibroblast activation^105^	No therapeutic inhibition validation
miR-21	Inhibition (indirect via Rb1)^106^	Ang II mouse model + primary cardiac fibroblasts + exosome analysis^106^	Downregulation reduces fibrosis, inflammation, and ECM gene expression^106^	Exosomal miR-21–mediated fibroblast activation and inflammatory signaling^106^	Indirect targeting; pleiotropic drug effects; limited mechanistic specificity^106^
miR-31^107^	Inhibition^107^	Human atrial tissue and myocytes, pacing-induced atrial fibrillation in goats, and mouse in vivo^107^	Silencing miR-31 restores dystrophin–neuronal nitric oxide synthase signaling, normalizes action-potential duration and rate adaptation, and may reduce susceptibility to atrial fibrillation^107^	miR-31–dystrophin–nNOS signaling axis^107^	Primarily targets electrical rather than fibrotic remodeling; direct antifibrotic efficacy, systemic delivery, and long-term safety remain unestablished.

The table highlights candidate microRNAs, proposed therapeutic strategies (upregulation or inhibition), experimental models, key antifibrotic or profibrotic findings, underlying mechanistic pathways, and major limitations.

Ang II = angiotensin II; ASPN = asporin; DYRK2 = dual-specificity tyrosine phosphorylation-regulated kinase 2; ECM = extracellular matrix; ISO = isoproterenol; miRNA = microRNA; nNOS = neuronal nitric oxide synthase; NFATc3 = nuclear factor of activated T cells, cytoplasmic 3; Rb1 = RB transcriptional corepressor 1; SOX9 = SRY-box transcription factor 9; SYK = spleen-associated tyrosine kinase; TAC = transverse aortic constriction.

Despite promising preclinical findings, significant challenges remain in translating these approaches clinically, particularly with respect to targeted delivery, specificity, and long-term safety.

Collectively, these studies demonstrate repeated convergence on a small number of signaling hubs, most notably TGF-β, NFAT, β-catenin, inflammatory pathways, and fibroblast activation programs. This suggests that future strategies may benefit from targeting shared regulatory nodes rather than individual microRNAs.

### Cell-based and regenerative strategies

Emerging approaches include direct fibroblast reprogramming, engineered extracellular vesicles, and immune-cell therapies designed to selectively target pathogenic fibroblast populations (Table 2).

**Table 2:** Recent Preclinical Studies Investigating Cell-Based and Regenerative Strategies to Target Cardiac Fibrosis

Technique	Study Design	Main Fibrosis-Related Finding	Key Limitation
Direct cardiac reprogramming of fibroblasts^108^	Chronic MI transgenic mouse model; tamoxifen-inducible MGTH overexpression in resident cardiac fibroblasts; validated in acute and chronic MI; single-cell RNA-seq + microarray for mechanism^108^	Approximately 2% of resident fibroblasts converted to iCMs; reduced fibrosis and improved contractility; antifibrotic effect driven by suppression of profibrotic fibroblast subpopulations and downregulation of Meox1, shifting fibroblasts toward a quiescent state^108^	Extremely low reprogramming efficiency (approximately 2%), meaning functional benefit likely relies on paracrine/transcriptional reprogramming rather than true regeneration; also dependent on genetic (transgenic) delivery, limiting translational feasibility
Microneedle patch delivering miR-29b-loaded HUCMSC exosomes^109^	Mouse LAD ligation MI model; gelatin microneedle patch implanted over infarct zone to locally deliver human umbilical cord MSC-derived exosomes loaded with miR-29b mimic; supported by in vitro TGF-β-stimulated murine cardiac fibroblast assays^109^	Reduced infarct size and fibrosis area, downregulated Col-I, Col-III, MMP-2, and MMP-9, alleviated inflammation; mechanistically linked to miR-29b restoration and likely suppression of TGF-β/Smad3-associated fibrotic signaling^109^	Translational feasibility is limited by the need for open-chest patch implantation and short-term validation in a small mouse model (28 d)
FAP-targeted CAR macrophages^110^	Mouse myocardial I/R model; bone marrow-derived macrophages were lentivirally engineered with a first-generation anti-FAP CAR; supported by in vitro phagocytosis assays, histology, RNA-seq, and 3-month follow-up^110^	FAP CAR-Ms selectively phagocytosed FAP + activated fibroblasts, reduced fibroblast abundance, decreased fibrosis, downregulated profibrotic genes including Col3a1, Tgfb2, periostin, Vcan and Timp1, and improved function with sustained benefit at 3 mo^110^	FAP is not fully specific to pathogenic fibroblasts, so broader fibroblast depletion could risk disrupting adaptive scar formation or repair; this was a proof-of-concept mouse study so human safety, antigen specificity, manufacturability, and optimal timing remain unresolved
CD248-targeted BBIR-T cell therapy against late-activated fibroblasts^111^	Mouse LAD ligation MI model with scRNA-seq, stereo-seq, flow cytometry, and immunostaining used to identify CD248 as a marker of late-activated fibroblasts (F-Act); human ischemic cardiomyopathy tissue validation^111^	Selective clearance of CD248^+^ F-Act fibroblasts after scar maturation reduced peri-infarct fibrosis expansion, limited ventricular remodeling, and improved systolic and diastolic function^111^	Therapeutic durability was limited, as the antifibrotic benefit weakened by 3 mo and likely requires repeat dosing; translation is also constrained by the complex 2-step delivery strategy
In vivo engineered FAP CAR macrophages via lipid nanoparticles^112^	Mouse myocardial I/R model; DOPS-doped macrophage-targeted LNPs carrying FAP CAR mRNA were given intravenously to generate CAR macrophages in vivo; in vitro phagocytosis assays, biodistribution analysis, fibrosis quantification, echocardiography, safety studies, and delayed-treatment experiments^112^	Generated functional FAP CAR macrophages that selectively phagocytosed FAP^+^ activated cardiac fibroblasts, reduced activated fibroblast abundance, decreased collagen III and overall fibrosis, and improved cardiac function at 2 wk and 2 mo; benefit was retained with delayed treatment^112^	Depends on repeated systemic lipid nanoparticle dosing; off-target organ accumulation, dose/cost scalability, and transient expression; FAP does not capture the full heterogeneity of activated fibroblasts
GDF15-preconditioned iPSC-MSC-derived exosomes^113^	Murine myocardial infarction model with intramyocardial injection of exosomes into the infarct border zone 30 min post-LAD ligation; complemented by in vitro TGF-β1–stimulated cardiac fibroblast model^113^	Reduced fibrotic area, collagen deposition, and myofibroblast activation (α-SMA) and improved cardiac function vs standard MSC-EXOs; suppressed fibroblast activation via repression of the MFAP4/ERK/Drp1 axis^113^	Strong efficacy relies on acute local delivery immediately post-MI, limiting clinical applicability; exosome heterogeneity and cargo complexity mean other bioactive components may contribute to effects.

The table summarizes the therapeutic technique, study design, principal fibrosis-related findings, and key translational limitations for each study.

α-SMA = alpha-smooth muscle actin; BBIR-T = bispecific immune receptor T; CAR = chimeric antigen receptor; CD248 = cluster of differentiation 248; Col-I = collagen type I; Col-III = collagen type III; Col3a1 = collagen type III alpha 1 chain; DOPS = 1,2-dioleoyl-sn-glycero-3-phospho-L-serine; Drp1 = dynamin-related protein 1; ERK = extracellular signal-regulated kinase; EXOs = exosomes; FAP = fibroblast activation protein; F-Act = late-activated fibroblasts; GDF15 = growth differentiation factor 15; HUCMSC = human umbilical cord mesenchymal stem cell; iCMs = induced cardiomyocytes; iPSC = induced pluripotent stem cell; I/R = ischemia/reperfusion; LAD = left anterior descending; LNPs = lipid nanoparticles; MCF = macrophage; Meox1 = mesenchyme homeobox 1; MFAP4 = microfibrillar-associated protein 4; miR-29b = microRNA-29b; MI = myocardial infarction; MMP-2 = matrix metalloproteinase-2; MMP-9 = matrix metalloproteinase-9; MSC = mesenchymal stem cell; RNA-seq = RNA sequencing; scRNA-seq = single-cell RNA sequencing; Smad3 = SMAD family member 3; TGF-β = transforming growth factor beta; TGF-β1 = transforming growth factor beta 1; Tgfb2 = transforming growth factor beta 2; Timp1 = TIMP metallopeptidase inhibitor 1; Vcan = versican.

Although these strategies demonstrate encouraging antifibrotic effects in preclinical models, challenges relating to delivery, specificity, scalability, and long-term safety continue to limit clinical translation. These limitations underscore why even highly promising cell- and gene-based approaches have not yet advanced beyond the early preclinical stage in cardiac fibrosis.

Notably, most approaches rely on highly controlled delivery conditions that are not currently scalable to clinical practice, limiting the translational potential of strategies that depend on precise exogenous manipulation. This challenge highlights the need for therapeutic approaches that can achieve greater biological specificity with clinically feasible delivery. Accordingly, future success may depend less on broadly suppressing fibrosis and more on selectively reprogramming pathologic fibroblast phenotypes.

## Clinical Evidence for Antifibrotic Therapies in Cardiovascular Disease

A notable feature of the clinical landscape is that most therapies evaluated to date were not developed as antifibrotic agents per se, but rather exert indirect effects on fibrotic remodeling, complicating the interpretation of observed benefits. Consistent with this, no therapy currently used in clinical practice directly targets myocardial fibrosis, despite its significant contribution to cardiovascular morbidity and mortality.^6^

### Heart failure

In HFrEF, neurohormonal antagonists remain the cornerstone of therapy according to the 2021 European Society of Cardiology guidelines.^114^ Recent studies suggest that sacubitril/valsartan may favorably influence cardiac remodeling, with improvements in left ventricular function and imaging markers of fibrosis.^115^ However, interpretation is limited by the modest sample size and semiquantitative assessment of fibrosis. Similarly, biomarker studies have reported changes in remodeling-related markers such as sST2 following sacubitril/valsartan treatment.^116^ Nevertheless, the absence of direct fibrosis assessment and reliance on surrogate endpoints mean that fibrosis-related benefits in HFrEF remain inferred rather than definitively established. A summary of key clinical studies evaluating fibrosis-targeting strategies in HFpEF is presented below in Table 3.

**Table 3:** Summary of Key Clinical Studies Evaluating Fibrosis-Targeting Strategies in HFpEF

Drug/Intervention	Population	Fibrosis Measurement	Key Outcomes	Key Limitation
Pirfenidone (PIROUETTE trial)^117^	Patients with HFpEF (LVEF ≥45%) and evidence of myocardial fibrosis on CMR (ECV ≥27%); randomized, double-blind, placebo-controlled phase 2 trial; 94 patients randomized^117^	Direct: cardiovascular magnetic resonance–derived myocardial extracellular volume, measured at baseline and 52 wk^117^	Reduced myocardial fibrosis, with a between-group difference in ECV of −1.21% vs placebo, and reduced NT-proBNP^117^	Small phase 2 trial, not powered for clinical outcomes; substantial treatment discontinuation because of adverse effects; findings show fibrosis regression, but whether this translates into meaningful long-term clinical benefit remains uncertain
Dapagliflozin^118^	100 patients with HFpEF and T2D; multicenter, double-blind, placebo-controlled trial; 12-mo follow-up^118^	Cardiac MRI-derived ECV at baseline, 6 and 12 mo^118^	Dapagliflozin reduced ECV more than placebo, suggesting regression of myocardial fibrosis; also reduced LVMI, NT-proBNP^118^	Small trial, restricted to diabetic HFpEF, no tissue-based fibrosis validation or collagen-turnover biomarkers, and short follow-up limits conclusions on long-term outcomes
Empagliflozin^119^	1084 patients from EMPEROR-Reduced and EMPEROR-Preserved; pooled HF population including HFrEF and HFpEF^119^	Serum collagen turnover biomarkers: PICP, PRO-C3, PINP, PRO-C6, C1M and C3M measured at baseline, 12 and 52 wk^119^	Empagliflozin reduced PICP by 5% at week 12 and 8% at week 52, and reduced PRO-C3 by 7% at week 52, suggesting reduced type I and III collagen synthesis^119^	Not HFpEF-specific and did not directly measure myocardial fibrosis by CMR, biopsy, or histology; circulating biomarkers may reflect fibrosis in other organs

Studies include both direct imaging-based assessments of myocardial fibrosis, primarily using cardiovascular magnetic resonance–derived extracellular volume (ECV), and indirect evaluation through circulating biomarkers of collagen turnover.

C1M = type I collagen degradation marker; C3M = type III collagen degradation marker; CMR = cardiac magnetic resonance; EMPEROR-Preserved = Empagliflozin Outcome Trial in Patients With Chronic Heart Failure With Preserved Ejection Fraction; EMPEROR-Reduced = Empagliflozin Outcome Trial in Patients With Chronic Heart Failure With Reduced Ejection Fraction; HFpEF = heart failure with preserved ejection fraction; HFrEF = heart failure with reduced ejection fraction; LVMI = left ventricular mass index; NT-proBNP = N-terminal pro–B-type natriuretic peptide; PICP = procollagen type I C-terminal propeptide; PINP = procollagen type I N-terminal propeptide; PIROUETTE = Pirfenidone in Patients With Heart Failure and Preserved Ejection Fraction; PRO-C3 = type III collagen formation marker; PRO-C6 = type VI collagen formation marker; T2D = type 2 diabetes.

Despite promising signals of fibrosis regression on imaging and biomarkers, current clinical evidence in HFpEF is largely exploratory and does not yet support the classification of these therapies as disease-modifying, given the absence of consistent associations between fibrosis reduction and clinical endpoints. Importantly, observed reductions in extracellular volume or collagen turnover markers have not been consistently linked to improvements in hard clinical outcomes such as hospitalization for heart failure or cardiovascular mortality.

### Post-MI remodeling

Clinical evidence for directly targeting fibrosis post-MI remains limited. The most explicit antifibrotic post-MI strategy is pirfenidone, but published patient outcome data are not yet available.^120^ Existing clinical evidence comes from indirect approaches, including molecular imaging of fibroblast activation,^121^ which enable in vivo quantification of fibrotic activity but do not directly evaluate therapeutic fibrosis regression. These findings highlight a persistent gap between the ability to measure fibrosis activity and the development of therapies that reliably regress established fibrosis in patients.

### Atrial fibrillation

Atrial fibrosis is recognized as a key contributor to the arrhythmogenic substrate underlying AF.^122^ Mechanistic studies of atrial remodeling highlight the role of paracrine signaling in directly linking fibrosis to arrhythmogenesis, with oxidative stress acting as an upstream modulator of atrial remodeling.^9^^,^^18^ Clinical trials targeting atrial fibrosis remain limited, with DECAAF II representing the only large evaluation of a fibrosis-guided intervention.^123^ Although DECAAF II tested magnetic resonance imaging–guided fibrosis ablation, the overall trial did not demonstrate superiority over conventional ablation for arrhythmia recurrence, although subgroup signals in lower-fibrosis patients warrant further investigation. Pharmacologic approaches remain investigational. The EDORA trial is evaluating whether dronedarone can attenuate left atrial fibrosis progression following ablation, although definitive fibrosis-related outcome data are not yet available.^124^ Biomarker studies further suggest that profibrotic mediators such as galectin-3 are associated with atrial remodeling and recurrence after ablation.^125^ Nevertheless, no therapy has yet been shown to reverse or substantially attenuate atrial fibrosis in patients.

### Other cardiovascular disease

Emerging studies in hypertensive heart disease, aortic stenosis, and diabetic cardiomyopathy suggest that myocardial fibrosis may be modifiable,^126^, ^127^, ^128^ although data remain limited and largely surrogate endpoint driven (Table 4).

**Table 4:** Clinical Studies Investigating Antifibrotic Therapies in Other Cardiovascular Disease Settings

Disease Setting	Therapy	Study Design	Fibrosis Endpoint	Main Finding	Limitation
Severe aortic stenosis after transcatheter aortic valve implantation, with high myocardial fibrotic burden^126^	Spironolactone ± dihydralazine^126^	Phase 3, randomized, open-label; n ≈ 300; ≥60 y^126^	Cardiac magnetic resonance imaging measurement of extracellular volume–derived matrix volume and late gadolinium enhancement^126^	Evaluates whether antifibrotic therapy reduces fibrosis at 12 mo postimplant^126^	No published results yet; open-label design introduces potential bias; reliance on imaging markers rather than direct tissue measurement; selection of only high-fibrosis patients limits applicability
Hypertensive heart disease with left ventricular hypertrophy^127^	Sacubitril and valsartan compared with valsartan alone^127^	Randomized, open-label, blinded-endpoint phase 2 trial; 78 patients with essential hypertension and left ventricular hypertrophy; 52-wk treatment period^127^	Cardiovascular magnetic resonance measurement of interstitial volume as a marker of diffuse interstitial fibrosis^127^	Sacubitril and valsartan produced greater reduction in interstitial volume than valsartan alone after 52 wk, despite similar blood pressure control^127^	Small phase 2 study; open-label treatment allocation; effect size was modest; 2 participants received mineralocorticoid receptor antagonists, which could also affect fibrosis
Diabetic kidney disease with heart failure and left ventricular ejection fraction of at least 40%^128^	Finerenone^128^	Phase 4, prospective, open-label, single-arm cardiac imaging trial; estimated 40 patients; 6 mo of treatment^128^	Cardiac magnetic resonance imaging measurement of extracellular volume fraction as a marker of diffuse myocardial fibrosis^128^	Evaluates whether finerenone reduces myocardial fibrosis and myocardial fat infiltration after 6 months of treatment^128^	No published outcome data; very small, estimated sample size; single-arm design means there is no control group; open-label design introduces potential bias; short follow-up period

## Limitations of Current Clinical Evidence

Despite growing interest in antifibrotic therapies, the clinical evidence base remains limited and heterogeneous. Across studies, fibrosis is most often assessed using cardiac magnetic resonance imaging-derived surrogate markers, such as extracellular volume or interstitial volume, which provide indirect measures of diffuse fibrosis and may not fully reflect true histologic regression.^117^, ^118^, ^119^^,^^127^^,^^128^ Furthermore, many studies are early phase and underpowered to assess major clinical outcomes, as demonstrated in trials evaluating pirfenidone, sacubitril and valsartan, and finerenone.^117^^,^^127^^,^^128^ In addition, observed reductions in fibrosis may be confounded by improvements in hemodynamic load or reverse remodeling, particularly in settings such as postvalve intervention following transcatheter aortic valve implantation.^126^ Finally, substantial heterogeneity in study populations, including differences in underlying disease, comorbidities, and baseline fibrotic burden, further limits cross-study comparison and the identification of consistent therapeutic benefit.^117^, ^118^, ^119^^,^^127^^,^^128^ Collectively, current evidence suggests that translational failure does not reflect the absence of biologically active therapies. Rather, failures arise from intervention in heterogeneous patient populations, incomplete assessment of fibrotic activity, inadequate consideration of disease stage, and insufficient ability to distinguish pathologic from adaptive fibroblast responses.

## Barriers to the Translation of Antifibrotic Therapies

Fundamental discrepancies between preclinical models and human cardiac fibrosis, including differences in disease chronicity, patient age, comorbidities, and molecular signaling, have contributed to the persistent failure of antifibrotic therapies to translate into clinical benefit.^75^ An overview of the key translational barriers limiting the development of antifibrotic therapies is summarized in Figure 4.

**Figure 4:**
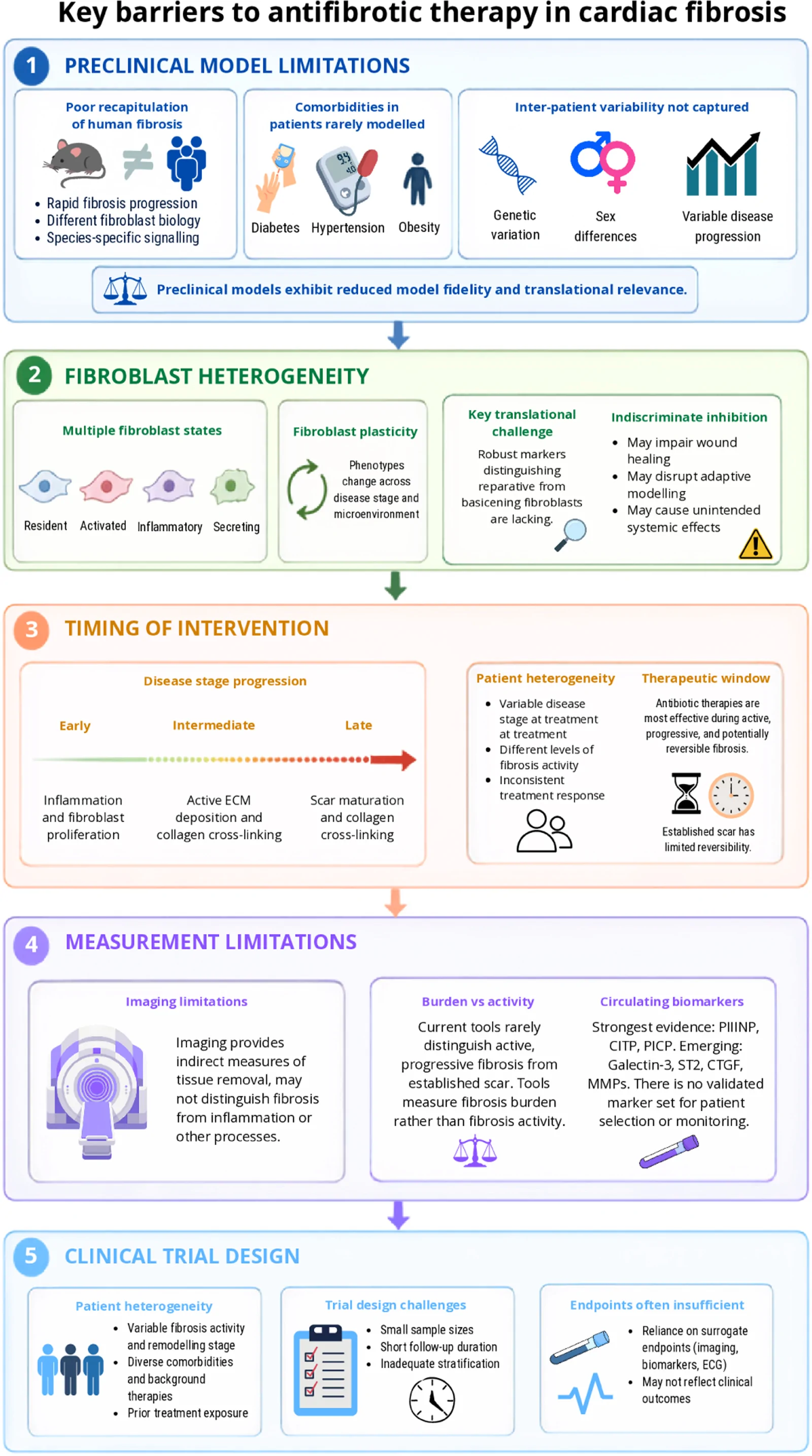
Barriers to the Translation of Antifibrotic Therapies Schematic overview highlighting key obstacles limiting clinical success, which collectively contribute to limited clinical benefit. CITP = collagen type I C-terminal telopeptide; CTGF = connective tissue growth factor; ECG = electrocardiogram; MMPs = matrix metalloproteinases; PICP = procollagen type I carboxy-terminal propeptide; PIIINP = procollagen type III N-terminal peptide; ST2 = suppression of tumorigenicity 2; other abbreviations as in Figure 2.

### Limitations of experimental models

A major barrier to translation is the inability of preclinical models to fully recapitulate the complexity of cardiac fibrosis observed in humans. Animal models often represent accelerated or simplified disease states and fail to capture the multifactorial and dynamic nature of human remodeling.^75^ Most studies are performed in young, otherwise healthy animals, whereas fibrosis typically develops in older patients with multiple comorbidities.^129^ Interpatient variation in disease burden, comorbidities, and prior treatment exposure further influences fibrotic processes and therapeutic response. Consequently, current models have limited predictive value for clinical efficacy.

### Fibroblast heterogeneity and context dependency

Indiscriminate inhibition of fibroblast activation may disrupt essential reparative processes and lead to unintended systemic effects.^74^ This lack of cellular specificity may be particularly problematic in settings such as post-MI remodeling, where excessive suppression of fibrotic responses could compromise structural integrity. A key challenge is the lack of markers and targeting strategies capable of distinguishing pathogenic from reparative fibroblast states across different stages of disease. These considerations emphasize the context-dependent nature of fibrosis and the difficulty of therapeutic targeting without inadvertently impairing beneficial repair.

### Timing of therapeutic intervention

The effectiveness of antifibrotic therapy is highly dependent on disease stage. In settings such as post-MI remodeling, fibrosis initially serves an essential reparative role, whereas established fibrosis may be less amenable to intervention.^130^ This distinction between adaptive and pathologic fibrosis further highlights the importance of disease stage when selecting antifibrotic interventions. This temporal dependency may contribute to inconsistent clinical trial outcomes, as patients are often enrolled at heterogeneous disease stages.

### Challenges in fibrosis assessment and biomarkers

Accurate assessment of cardiac fibrosis remains a major obstacle. Imaging approaches such as extracellular volume quantification and late gadolinium enhancement provide indirect measures of remodeling and may not reliably distinguish fibrosis from inflammation. Circulating biomarkers offer a more practical approach for repeated assessment. Emerging proteomic approaches further suggest that broader biomarker profiling may capture inflammatory and matrix-related signals relevant to the progression of fibrotic remodeling.^131^ However, among a wide panel of circulating biomarkers, only markers of collagen synthesis and degradation show consistent associations with myocardial fibrosis, whereas most other candidates demonstrate weak, inconsistent, or absent relationships.^21^ The absence of validated fibrosis-specific biomarkers limits patient selection, monitoring of treatment response, and comparison across studies.

### Imaging fibrosis vs fibrotic activity

Current cardiac imaging methods mainly quantify established fibrosis. Late gadolinium enhancement cardiovascular magnetic resonance identifies focal replacement fibrosis, whereas T1 mapping and extracellular volume quantification are better suited to assessing diffuse interstitial fibrosis.^132^ In contrast, fibroblast activation protein inhibitor positron emission tomography targets fibroblast activation protein expressed by activated fibroblasts and may therefore provide a marker of ongoing fibroblast activation and fibrotic activity rather than established ECM accumulation alone.^133^^,^^134^ This can potentially improve patient selection and therapeutic monitoring. Collectively, these limitations support a shift from measuring accumulated fibrosis burden towards identifying ongoing fibrotic activity and therapeutically relevant fibroblast states, as summarized in Figure 5.

**Figure 5:**
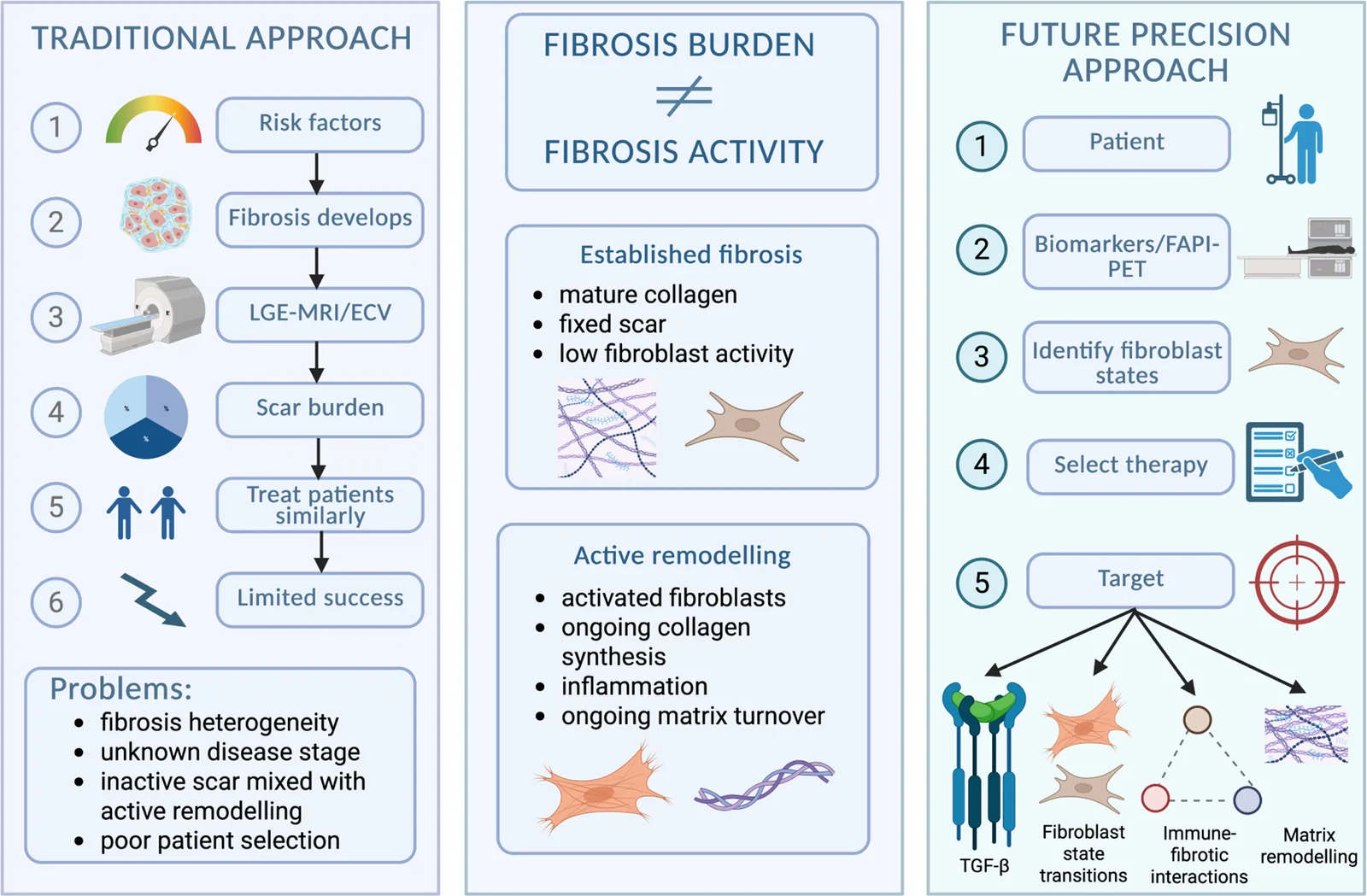
From Fibrosis Burden to Fibrotic Activity: A Precision-Medicine Framework for Antifibrotic Therapy Traditional approaches primarily quantify established scar burden using imaging markers such as late gadolinium enhancement and extracellular volume but may not distinguish inactive fibrosis from ongoing remodeling. Integrating fibroblast activation protein inhibitor positron emission tomography (FAPI-PET), circulating biomarkers, and single-cell or spatial profiling could identify active fibroblast states and enable selection of therapies targeting transforming growth factor-beta (TGF-β) signaling, fibroblast-state transitions, immune-fibrotic interactions, or extracellular matrix remodeling. ECV = extracellular volume; LGE-MRI = late gadolinium enhancement-magnetic resonance imaging.

### Safety and off-target effects

Broad systemic inhibition of such profibrotic signaling pathways can carry an inherent risk of off-target effects,^81^^,^^135^ which may restrict the therapeutic window of antifibrotic interventions and limit the feasibility of long-term antifibrotic therapy in chronic conditions such as persistent AF, heart failure, and hypertension. In addition, fibrotic signaling is highly redundant and involves multiple interacting pathways, including TGF-β and RAAS,^13^, ^14^, ^15^ such that inhibition of a single pathway (a monotherapy) may be insufficient to achieve meaningful antifibrotic effects. This challenge is further compounded by the lack of cardiac- or fibroblast-specific delivery strategies, which limits the ability to selectively modulate pathogenic fibroblast activity without affecting other tissues or physiological repair processes. An additional translational consideration is whether durable antifibrotic efficacy may require simultaneous treatment of the underlying disease driver. Persistent cardiomyocyte injury, hemodynamic overload, metabolic dysfunction, or inflammation could continue to provide profibrotic stimuli despite direct inhibition of fibrotic pathways. Conversely, treatment of the primary disease process may not be sufficient once fibrosis has become self-sustaining. Rational combination strategies targeting both the initiating pathologic stimulus and active fibrotic remodeling may therefore warrant consideration, with the optimal approach likely to depend on disease mechanism and stage.

### Translational trial design limitations

Many clinical studies are limited by small sample sizes, short follow-up durations and reliance on surrogate endpoints, which reduce the ability to detect meaningful effects on long-term clinical outcomes. Evidence from recent clinical and preclinical pulmonary fibrosis studies supports the concept that antifibrotic therapies may be more effective when used in combination, as combined regimens outperform monotherapy in improving survival and suppressing inflammatory and fibrotic pathways.^136^^,^^137^ This suggests that evaluation of antifibrotic therapies in isolation may fail to capture additive or synergistic effects that could arise when combined with established disease-modifying treatments.

The key translational barriers and emerging strategies to overcome them are summarized in the Central Illustration.

**Central Illustration:**
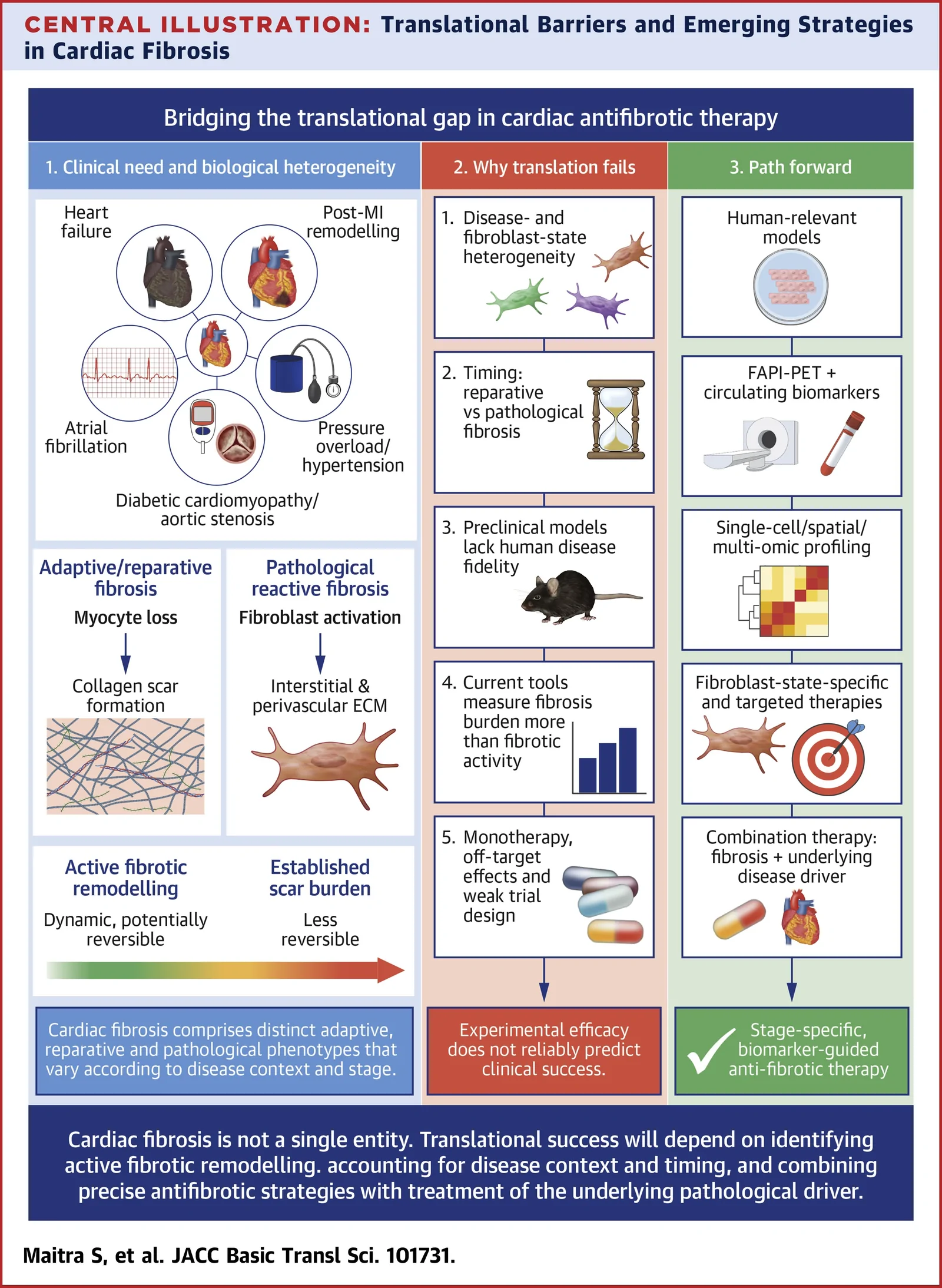
Translational Barriers and Emerging Strategies in Cardiac Fibrosis Overview of the key barriers limiting clinical translation of antifibrotic therapies and emerging strategies to enable more precise, stage-specific treatment.

## Emerging Strategies and Future Perspectives

Future progress will require more predictive human-relevant models, including engineered heart tissues, organoids, and heart-on-chip systems that better capture fibroblast heterogeneity and chronic disease biology. Precision medicine approaches integrating single-cell and spatial profiling with multi-omic data sets, advanced imaging, and circulating biomarkers may improve patient stratification according to fibrosis stage, disease context, and underlying molecular drivers.^138^ Integration across transcriptomic, epigenomic, proteomic, and metabolomic layers may further help define therapeutically relevant fibroblast states and multicellular signaling networks that are not captured by individual modalities alone.^138^ Artificial intelligence may complement these approaches by integrating high-dimensional molecular and clinical data sets to support target identification, drug discovery and patient stratification, although prospective biological and clinical validation will remain essential. In parallel, targeted delivery systems and rational combination therapies may enhance efficacy while minimizing off-target effects. Future trials should incorporate disease-stage-specific enrollment, longer follow-up, validated fibrosis endpoints, and hard clinical outcomes.

Moreover, emerging cell-targeted approaches, including CAR-based strategies, suggest that future antifibrotic therapies may move beyond conventional inhibition of individual profibrotic pathways toward selective modulation or elimination of pathogenic fibroblast populations and their interactions with the surrounding microenvironment. Fibroblast activation protein-targeted chimeric antigen receptor T-cell therapy has demonstrated the feasibility of transiently targeting activated cardiac fibroblasts in vivo, reducing fibrosis and restoring cardiac function in a preclinical model of heart failure.^139^ Likewise, IL-11, a fibroblast-enriched downstream mediator of TGF-β signaling, promotes ERK-dependent fibrogenic protein synthesis and may offer a more selective antifibrotic target than upstream TGF-β inhibition.^15^^,^^115^ More recently, integrated single-cell and spatial transcriptomic analyses of human failing hearts identified FAP^+^/POSTN^+^ fibroblast populations driven by C-C motif chemokine receptor 2^+^ macrophage-derived IL-1β signaling, highlighting immune–fibroblast crosstalk as a therapeutically targetable mechanism.^65^ Together, these strategies have the potential to move the field beyond broad, nonspecific approaches toward precise, stage-appropriate, and patient-tailored antifibrotic therapies.

## Conclusions

Excessive ECM deposition driven by cardiac fibroblasts represents a unifying feature of pathologic cardiac remodeling across diverse cardiovascular disease states, contributing to impaired tissue compliance, disrupted electrical signaling, and progression to heart failure. Advances in the understanding of cardiac fibroblast biology and profibrotic signaling pathways have established fibrosis as a biologically plausible therapeutic target. However, despite substantial progress at the mechanistic and preclinical levels, the translation of antifibrotic therapies into consistent clinical benefit has been limited.^75^

Current evidence is constrained by limitations of experimental models, fibroblast heterogeneity, challenges in fibrosis assessment, temporal dependence of therapeutic efficacy, and weaknesses in clinical trial design. The limited success of antifibrotic therapies highlights the need to move beyond broadly targeted approaches toward more precise and biologically informed strategies.

Ultimately, the central challenge is not simply identifying therapies capable of reducing fibrosis, but determining when, where, and who antifibrotic interventions are most likely to be effective. Addressing this challenge will be essential for translating mechanistic advances into therapies capable of delivering meaningful and sustained benefit for patients with cardiovascular disease. To achieve this, the field should prioritize: 1) humanized preclinical models that better predict clinical efficacy; 2) fibroblast state-specific targeting strategies that spare reparative functions; 3) validated direct or surrogate endpoints for fibrosis regression; and 4) innovative trial designs incorporating combination therapies, precision stratification, and hard clinical outcomes. These steps will be critical to realizing the therapeutic potential of antifibrotic interventions in heart disease. Ultimately, successful translation will depend on moving beyond the traditional view of fibrosis as a static measure of scar burden and instead targeting active, context-dependent fibrotic remodeling. Bridging the gap between mechanistic insight and clinical efficacy will require precision, stage-specific intervention strategies tailored to disease biology, fibroblast state, and patient-specific risk. In this context, the key challenge is no longer whether fibrosis can be modified, but how to identify the right patients, at the right stage of disease, for the right antifibrotic intervention.

## Funding Support and Author Disclosures

This work was supported by British Heart Foundation Fellowship FS/SBSRF/22/31033 (to S.R.). The authors have reported that they have no relationships relevant to the contents of this paper to disclose.
